# Analysis of Snail-1, E-Cadherin and Claudin-1 Expression in Colorectal Adenomas and Carcinomas

**DOI:** 10.3390/ijms13021632

**Published:** 2012-02-02

**Authors:** Michala Bezdekova, Svetlana Brychtova, Eva Sedlakova, Katerina Langova, Tomas Brychta, Kamil Belej

**Affiliations:** 1 Laboratory of Molecular Pathology, Department of Clinical and Molecular Pathology, Faculty of Medicine and Dentistry, Palacky University, Hnevotinska 3, 775 15 Olomouc, Czech Republic; E-Mails: michala.bezdekova@seznam.cz (M.B.); Pomnenka77@seznam.cz (E.S.); 2 Department of Medical Biophysics, Faculty of Medicine and Dentistry, Palacky University, Hnevotinska 3, 775 15 Olomouc, Czech Republic; E-Mail: langova@tunw.upol.cz; 3 Department of Recreology, Faculty of Physical Culture, Palacky University, tr. Miru 115, 771 11 Olomouc, Czech Republic; E-Mail: tomas.brychta@volny.cz; 4 Department of Urology, Central Military Hospital, U Vojenské nemocnice 1200, 169 02 Prague 6, Czech Republic; E-Mail: kamil.belej@uvn.cz

**Keywords:** Snail-1, E-cadherin, claudin-1, adenocarcinoma, adenoma, immunohistochemistry

## Abstract

We report the expression of Snail-1, E-cadherin and claudin-1 by indirect immunohistochemistry in colonic neoplasia. Snail-1 is a zinc finger transcription factor expressed in cells that already have undergone almost complete epithelial-mesenchymal transition (EMT) and have already evaded from the tumor. The main mechanism by which Snail induces EMT is downregulation of E-cadherin, of which expression was shown to be frequently downregulated in many different types of tumors, where it accompanies the invasiveness and metastatic behavior of malignant cells. Moreover, Snail-1 may downregulate the expression of claudin-1, a cell-cell adhesion protein which plays a likely role in progression and dissemination during tumorigenesis. Snail-1 was expressed in both carcinoma and adenoma cells with histologically normal epithelium in the mucosa, adjacent to the tumors, without significant differences, and predominant strong intensity of staining. Statistically significant differences were revealed between normal and tumorous epithelium (*p* = 0.003) at the subcellular level, where the shift of the protein to the cytoplasm with combined cytoplasmic/nuclear or pure cytoplasmic expression was observed. E-cadherin expression was present in 100% of cases of both adenocarcinomas and adenomas, with prevailing strong membranous immunoreactivity and no differences between protein expression in tumors and normal mucosa. Predominating strong positivity of claudin-1 was detected in tumor cells of adenocarcinomas and adenomas. Marked differences were seen in protein localization, where membranous staining, typical for nontumorous epithelium, changed to combined membranous/cytoplasmic expression in adenocarcinomas (*p* = 0.0001) and adenomas (0.0002), in which cytoplasmic shift was associated with a higher degree of dysplasia. Furthermore, membranous/cytoplasmic localization was more frequent in the carcinoma group (87%) in comparison with adenomas (51%) (*p* = 0.0001). We conclude that dystopic subcellular localizations of Snail-1 and claudin-1 may participate in changes of cellular morphology and behavior which might be associated with altered effectory pathways of proteins and thus substantially contribute to the cancer development.

## 1. Introduction

In recent years, research has shown that epithelial-mesenchymal transition (EMT) offers a relevant point for research. EMT refers to the process in which an epithelial cell disengages from its parent tissue by losing mediators of homotypic and/or heterotypic cell-cell interactions in exchange for morphology and adhesion marker profiles, consistent with a mesenchymal cell. It is regarded as the first necessary step for invasion and metastasis [1]. EMT is important in many developmental processes, playing crucial roles in the formation of the body plan and in the differentiation of multiple tissues and organs. Recently, a role for EMT in the process of cancer metastasis has been postulated. In carcinomas, aberrant activation of the process of EMT, by which cancer cells lose their polarity and are converted to a mesenchymal phenotype, is a late event in tumor progression; when cancer cells at the invasive front of primary tumors undergo a phenotypic conversion to invade and metastasize through the circulation and generate a metastatic lesion. The vast majority of known signaling pathways have been implicated in the regulation of EMT, from which a central role in this process is played by the transcriptional repressor Snail-1 [2]. Besides their involvement in EMT, Snail family members have been involved in a variety of other processes, such as apoptosis and left-right asymmetry. The Snail family includes Snail-1 (Snail), Snail-2 (Slug) and Snail-3 (Smuc), and has been shown to be over-expressed in a wide variety of human malignancies including oral [3], breast [4], hepatocellular [5], gastric [6], colon and skin carcinomas [7]. Snail-1 is a zinc finger transcription factor expressed in cells that already have undergone almost complete EMT and have already evaded from the tumor [8]. The observations presented so far associate Snail-1 with invasion, metastasis and poor prognosis [9]. The mechanism by which Snail-1 influences these different cellular processes remains largely unresolved. The main mechanism by which Snail-1 induces EMT is downregulation of E-cadherin [10,11] by direct repression of the protein through binding to E-boxes in the E-cadherin promoter [12].

A loss of E-cadherin expression is considered to be a hallmark of EMT. E-cadherin, a key transmembrane molecule cell adhesion protein, is a member of a family of functionally related transmembrane glycoproteins that mediate Ca^2+^-dependent intercellular adhesion. The adhesion is mediated via interaction with adjacent cells through their *N*-terminal ectodomains. The cytoplasmic terminal tail of E-cadherin links specifically to β-catenin that binds directly to cytoskeletal actin, which helps to assemble epithelial cell sheets. A loss of its expression or function diminishes cell-cell contacts, which leads to impaired intercellular signaling, but not to direct tumor transformation. E-cadherin expression was shown to be frequently downregulated in many different types of tumors, where it accompanies the invasiveness and metastatic behavior of malignant cells [13].

Besides adherent junctions (AJs), cell-cell adhesions are maintained through tight junctions (TJs). In contrast to the role of AJs, the role of TJ proteins in cancer is less well understood. TJs are the most apical cell-cell contacts and are important for a barrier function that regulates the passage of ions, water, and macromolecules, together with a fence function that maintains cell polarity. A number of integral membrane proteins associated with TJs have been identified, including the claudin family consisting of at least 24 members, of which claudin-1 was also recently identified as a probable target of β-catenin/Tcf signaling. In colorectal cancer (CRC), different data have been published concerning claudin-1 expression in cancer cells. On the one hand, claudin-1 upregulation was described at both RNA and protein levels [14,15]. On the other hand, reduced claudin-1 expression was shown to correlate with poor differentiation, disease recurrence and poor patient survival [16]. It has been shown that Snail-1 may downregulate the expression of claudin-1 [17]. Another study emphasizes the role of E-cadherin expression and localization in the claudin-1 dependent regulation of EMT, suggesting that claudins play a role in progression and dissemination during tumorigenesis [15].

The goal of this study is to report the expression of Snail-1, E-cadherin and claudin-1 in colonic neoplasia, as colorectal tumors provide an excellent system to study the genetic alterations involved in the development of common human neoplasms [18]. Despite the fact that many data concerning similar topics have been published up to now, their results often vary. Moreover, little is known about stromal protein expression and its impact on colon cancerogenesis.

## 2. Results

### 2.1. Snail-1

**Figure 1:**
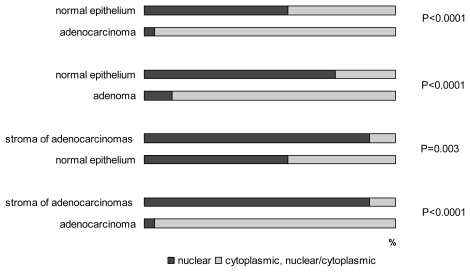
Cellular localization of Snail-1.

The protein was expressed in high levels without significant differences between carcinomas, adenomas and histologically normal epithelium in the mucosa adjacent to the tumors. Snail-1 protein was also evaluated in adenocarcinomas according to their grade. Interestingly, 10% of grade 3 tumors showed a decrease in expression from strong to weak (*p* = 0.009). Within nontumorous colon epithelium, Snail-1 was localized predominantly in the base of crypts; superficial areas exhibited weaker intensity or were negative. Statistically significant differences were revealed between normal and tumorous epithelium (*p* = 0.003) at the subcellular level, where the shift of the protein to the cytoplasm with combined cytoplasmic/nuclear or pure cytoplasmic expression was observed (Figure 1). In nontumorous cells of the epithelium, pure nuclear positivity predominated (57% of cases), while combined cytoplasmic/nuclear was detected in 34% and cytoplasmic in only 5% of cases. In carcinomas and adenomas, combined cytoplasmic/nuclear expression prevailed (64% of cases), pure nuclear expression was observed in 4%, and 32% of cases exhibited only cytoplasmic protein distribution.

**Figure 2:**
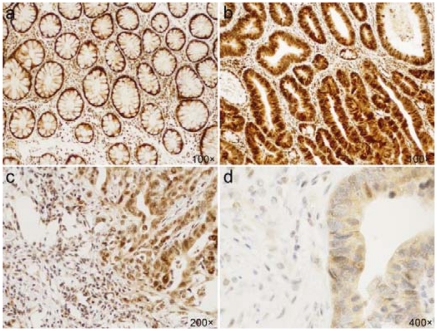
Immunohistochemical staining of Snail-1. (**a**) Nuclear expression in normal colonic epithelium; (**b**) Combined nuclear/cytoplasmic expression, with predominating nuclear localization in adenoma; (**c**) Combined nuclear/cytoplasmic expression in adenocarcinoma; (**d**) Purely cytoplasmic expression in adenocarcinoma.

Moreover, Snail-1 was detected in the stromal elements fibroblasts, endothelial cells and macrophages (Figure 2). Stromal elements in adenocarcinomas exhibited statistically higher levels (*p* < 0.0001) of nuclear Snail-1 positivity compared with the adjacent nontumorous stromal elements. Stroma of adenocarcinomas expressed significantly higher levels of Snail-1 when correlated with stroma of adenomas (*p* = 0.012). Interestingly, stroma of mucosa adjacent to adenocarcinomas showed higher numbers of Snail-1 positive elements when compared to normal mucosa close to adenomas (*p* = 0.031). There were no differences between stroma of adenomas and normal mucosa.

### 2.2. E-cadherin

**Figure 3:**
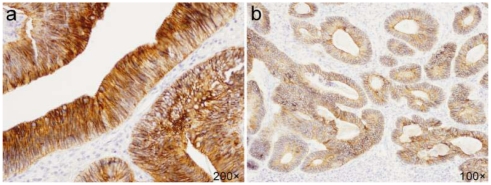
Immunohistochemical staining of E-cadherin. (**a**) Strong membranous expression in adenoma; (**b**) Moderate membranous expression in adenocarcinoma.

E-cadherin expression was present in 100% of cases of both adenocarcinomas and adenomas, with prevailing strong immunoreactivity (Figure 3). There were no differences between protein expression in tumors and normal mucosa. E-cadherin expression in adenocarcinomas was detected to be mostly strong in G2 and G3 tumors. G1 tumors covered significantly more patients with moderate expression (*p* = 0.026). The analysis did not show statistically significant differences in E-cadherin expression between neoplastic cells and normal epithelium. E-cadherin was localized on cell membranes and no changes in subcellular localization were found.

### 2.3. Claudin-1

**Figure 4:**
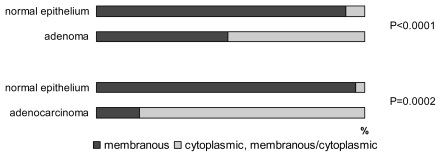
Cellular localization of claudin-1.

In tumor cells of adenocarcinomas and adenomas, prevailing strong positivity of claudin-1 was detected, without significant quantitative differences between the two groups. Claudin-1 was expressed in significantly higher levels in adenomas (*p* = 0.034) and adenocarcinomas (*p* = 0.005), comparing with normal epithelium. No differences were observed concerning the grade of carcinomas. Marked differences were seen in protein localization (Figure 4), where membranous staining, typical for nontumorous epithelium, changed to combined membranous/cytoplasmic expression in adenocarcinomas (*p* = 0.0001) and adenomas (0.0002), where cytoplasmic shift was associated with a higher degree of dysplasia. Furthermore, membranous/cytoplasmic localization was more frequent in the carcinoma group (87%) in comparison with adenomas (51%) (*p* = 0.0001).

**Figure 5:**
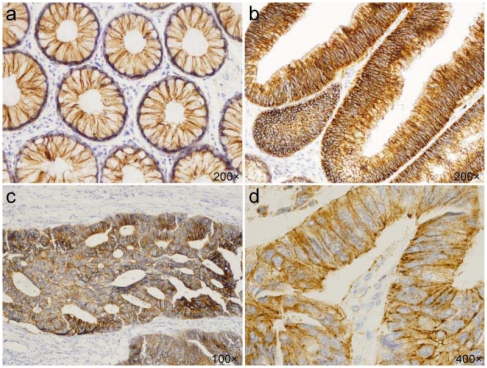
Immunohistochemical staining of claudin-1. (**a**) Membranous expression in normal epithelium; (**b**) Strong membranous expression in adenoma; (**c**) Combined membranous/cytoplasmic expression in adenocarcinoma; (**d**) Cytoplasmic expression in adenocarcinoma.

In stromal elements, claudin-1 was not regularly present (Figure 5). In all groups (benign mucosa, adenomas, adenocarcinomas), there were negative cases. In adenocarcinomas, however, a significant increase in the number of positive elements was observed compared to normal mucosa (*p* < 0.012).

## 3. Discussion

The purpose of this study was to report the changes of immunohistochemical expression and their localization of Snail-1, E-cadherin and claudin-1, proteins substantially involved in EMT of colon mucosa. EMT refers to the process whereby an epithelial cell disengages from its parent tissue by losing mediators of homotypic and/or heterotypic cell-cell interactions in exchange for morphology and adhesion marker profiles consistent with a mesenchymal cell. Generally, as EMT promotes invasion of cancer cells and metastasis, it is associated with worse prognosis for cancer patients [1]. However, despite recent advances, much remains unknown about the EMT program in cancer progression and metastasis, because cancer is a complex and multistep process, and EMT represents only a part of the process of tumor progression. Snail-1 is a transcriptional factor whose overexpression was shown to be important in intestinal tumorigenesis [19]. The Snail family is activated by multiple signaling pathways, including Wnt/β-catenin, TGF-β (transforming growth factor β), TNF-α (tumor necrosis factor α), RAS, ILK (integrin-linked kinase), NF-κB (nuclear factor κ-light-chain-enhancer of activated B cells), HIF (hypoxia-inducible factor), AKT activation and EGFR (epidermal growth factor receptor) signaling [20,21]. Varying data have been published concerning Snail expression in cancer cells and nontumorous epithelium. While Franci *et al.* (2009) described the protein only in carcinoma cells [8], Zhu *et al.* (2010) found Snail-1 expression in normal epithelium [22]. Similarly, in our study, the protein was detected in normal colon epithelium, where it was mostly bound to crypts. Thus, we concluded that Snail-1 may not necessarily be associated with malignant transformation, but might act by maintenance of a balance between cell proliferation, differentiation and apoptosis. Snail-1 is typically localized in the nucleus. However, the subcellular localization and stability are sensitive to Ser/Thr phosphorylation, when after being phosphorylated, Snail-1 is translocated to the cytosol, where it is not active and is subsequently degraded [8,23]. Phosphorylated Snail-1 is much less active as a repressor of E-cadherin transcription or as an activator of the expression of mesenchymal genes. When phosphorylated, Snail-1 retains the ability to bind DNA, so phosphorylation seems to block its access to the target promoter. Phosphorylation of Snail-1 is controlled by cellular attachment to the ECM, so extracellular environment may regulate its activity [23]. However, cytoplasmic localization of the protein might not be definitively associated with loss of its function. The possibility that cytoplasmic Snail-1 plays a positive role in the acquisition of migratory abilities by tumor cells cannot be totally discarded. We demonstrated increasing Snail-1 expression in stromal fibroblasts, macrophages and endothelial cells in adenomas and adenocarcinomas, confirming that such expression may play a substantial role in cancer progression. The mechanism by which Snail-1 in stroma may influence tumor growth is not fully understood. One possible explanation is immunosuppression through immunosuppressive cytokines, regulatory T cells, impaired dendritic cells and cytotoxic T lymphocyte resistance [24]. The presence of Snail-1 immunoreactive cells in the stroma may be an informative indicator of prognosis of colon tumors. It has been shown that intratumoral injection with a Snail-specific monoclonal antibody inhibits tumor growth and metastasis followed by an increase of tumor-specific tumor-infiltrating lymphocytes and systemic immune response [25]. Snail-1 is one of the strongest repressors of E-cadherin through binding to E-boxes in the E-cadherin promoter [10,11]. E-cadherin is important for epidermal intercellular adherence and loss of E-cadherin-mediated cell adhesion and is one rate-limiting step in the progression from adenoma to carcinoma [26,27]. Dysfunction or disruption of cell adhesion molecules also accompanies the invasiveness and metastatic behavior of malignant cells, when loss of E-cadherin consistently occurs at sites of EMT during cancer development. E-cadherin downregulation is commonly described in colorectal adenocarcinomas. However, in our study we revealed neither a decrease in protein levels nor its cytoplasmic shift from the membrane, and the protein expression did not differ from nontumorous epithelium.

Considering our findings, E-cadherin presence in CRC either caused no alteration of AJs or intercellular signaling may be at least partly E-cadherin independent. For example, Snail-1 can functionally interact with catenin and directly enhance the activation of Wnt signaling, thus establishing a positive feedback loop for Wnt-dependent transcription [28]. It has also been documented that Wnt and EGFR signaling pathways cross-talk mutually, and transactivation of each other has been described, so activation of catenin with a downstream effector of Wnt pathway can be mediated via EGFR. Also despite the fact that previous studies demonstrated a considerable inverse correlation between E-cadherin and Snail-1 mRNA levels in most epithelial tumor cell lines [3,7,10,11], Domínguez *et al.* (2003) reported simultaneous expression of E-cadherin and Snail-1 when detected in similar protein levels [23]. However, these facts deserve further investigation.

Snail-1 is a known suppressor of TJ proteins, of which claudin-1 was involved in our study. TJs belong to key molecular components governing cellular adhesion and polarity. Since in neoplastic cells, the expression of intestinal epithelial TJ proteins frequently exhibit structural and functional altrations, they might contribute to neoplastic progression via induction of epithelial dedifferentiation [29,30].

Differing data have been published concerning claudin-1 expression in colon tumors, describing both downregulation as well as overexpression of the protein. More frequently increased protein levels were found in adenomas and adenocarcinomas [15,31,32]. Low expression of claudin-1was correlated with higher tumor grades, acquisition of a metastatic phenotype and poor patient survival. Moreover, decreased claudin-1 expression has even been suggested to be a stronger predictor of recurrence and survival [16]. It is not well understood which factors, apart from Snail-1, are involved in dysregulation of claudin-1 expression. Growth factors EGF and TGF-beta may upregulate the protein levels. Claudin-1 was also identified as a target of beta-catenin/Tcf signaling pathway [15]. In our study, immunohistochemistry confirmed elevated expression level of claudin-1 both in adenomas, in particular adenomas with severe dysplasia, as well as in CRC, when compared to normal adjacent mucosa. We did not confirm loss of protein expression in G3 adenocarcinomas. As claudins regulate paracellular transport, they are usually found at the cell membrane. In concordance with other studies, we found predominantly a circumferential membranous pattern of staining for claudin-1 in normal colonic epithelium. However, in tumor lesions, claudin-1 exhibited dysregulated subcellular localization with moving away from its typical membranous position. Claudin-1 remained located on the cytoplasmic membranes mainly in adenomas without or with mild dysplasia, whereas the shift to the cytoplasm with cytoplasmic or membranous/cytoplasmic expression was observed in high-grade dysplastic adenomas and carcinomas. However, a minority of the tumors preserved cytoplasmic staining as well. We did not confirm the nuclear localization of claudin-1 [15]. The importance of claudin-1 mislocalization is not fully understood. Cytoplasmic claudin-1 localization is associated with induction of EMT, resulting in increased cell motility and metastatic potential. Loss of membranous expression may also stimulate tumor growth non-specifically, when alteration of TJ-regulated permeability may allow increased diffusion of nutrients and other factors critical for tumor growth and survival. Furthermore, claudins have been reported to recruit and promote the activation of MMP-2 (matrix metalloproteinase-2), suggesting potential involvement in invasion and metastasis.

Little is known about mechanisms affecting subcellular claudin-1 localization. It has been reported that protein kinases such as protein kinase A (PKA) and protein kinase C (PKC) are important for the regulation and cytoplasmic localization of claudin-1 expression [33,34]. The significance of claudin-1 in the nucleus is unclear. It is known that the nuclear expression of other TJs can either inhibit proliferation, or on the other hand may correlate with oncogenic transformation and proliferation. Only few data have been published concerning claudin expression in stromal elements. Claudin-1 expression has been described in immune cells, monocytes and lymphocytes, in association with their activation [35]. Claudin-1was detected in endothelial cells. Both decrease of its expression or overexpression may be associated with altered permeability [36,37].

We found the protein in endothelial cells, macrophages and lymphocytes, with only scattered elements being present within normal epithelium. An increased number of positive cells were found in adenomas and especially in carcinomas. It is not clear whether such expression influences tumor growth positively or negatively.

## 4. Material and Methods

### 4.1. Immunohistochemistry

Archival cases of grade 1 to 3 colon carcinomas from 120 consecutive patients and 42 samples of adenomas were retrieved from the archives of the Department of Clinical and Molecular Pathology, Faculty of Medicine and Dentistry, Palacky University Olomouc between 2007 and 2010. First, the corresponding H&E slides were reviewed by a pathologist to confirm the diagnosis and adequacy of material. All selected tissue samples were formalin fixed and paraffin embedded.

Immunohistochemistry for Snail-1, E-cadherin and claudin-1 was performed on 5-μm paraffin-embedded colorectal cancer tissue section. The slides were stained with antibodies: Snail-1 (rabbit polyclonal, Ab 85931, 1:150, Abcam), E-cadherin (mouse monoclonal, M3612, 1:50, Dako) and claudin-1 (mouse monoclonal, sc-81796, 1:500, Santa Cruz Biotechnology, Inc.) by an indirect immunohistochemical method using the BenchMark XT immunostainer (Ventana Medical Systems, Inc.).

The quality and intensity of staining as well as cellular localization were evaluated in tumor cells and the surrounding tumor stroma. Moreover, normal epithelium and its surrounding stroma were also assessed in each sample. Evaluation of protein expression was done by a modified H-score, in which both intensity and proportion of staining were categorized from 0 to 3.

Cell positivity was multiplied by intensity of staining to form a multiplicative score. The cases were sorted into four subgroups: Hscore 0 referred to negative expression; Hscore 12 to weak expression; Hscore 35 to moderate expression; and Hscore 69 to strong expression.

### 4.2. Statistical Analysis

The staining of protein expression in neoplastic cells was compared to that of normal epithelium and tumor grade. Furthermore, tumor stroma was compared to the surrounding stroma of normal epithelium and protein levels were also compared between adenomas and adenocarcinomas. The assessment was evaluated by Fisher’s exact test. Associations between binary categories were analyzed using the chi-square test. *P*-values of 0.05 or less were considered to be statistically significant.

## 5. Conclusions

In summary, we demonstrated that not only overexpression of both Snail-1 and claudin-1 is involved in transformation of colonic epithelium, but also changes in subcellular localizations, when predominantly cytoplasmic Snail-1 and claudin-1 expression might be important for malignant transformation and might substantially contribute to the cancer development. Our findings deserve further morphological as well as functional investigation.
